# Population Data Centre Profile - The Western Australian Data Linkage Branch

**DOI:** 10.23889/ijpds.v4i2.1138

**Published:** 2020-03-11

**Authors:** S Hodges, T Eitelhuber, A Merchant, J Alan

**Affiliations:** 1 The Western Australian Department of Health, Data Linkage Branch

## Abstract

Established in 1995, the Western Australian Data Linkage Branch (DLB) is Australia’s longest running data linkage agency. The Western Australian Data Linkage System (WADLS) employs an enduring linkage model spanning over 60 data collections supported by internally developed and supported software and IT infrastructure. DLB has delivered, and continues to deliver, a range of significant data linkage innovations, many of which have been adopted elsewhere. A current restructure within the Western Australian Department of Health (which we will refer to as the Department of Health) will provide an improved funding model geared toward addressing issues with staff retention, capacity and customer service, as well as fostering improvements to data management, governance and availability. Research using linked data provided by DLB has been used in over 800 projects resulting in over 2350 publications and outcomes for policy development, service delivery and public health. Demand continues to grow for data linkage services and with the Department of Health’s bolstered commitment to resourcing, DLB looks forward to a future for data linkage in Western Australia that is sustainable, high quality, efficient, and safe.

## Introduction

As Australia’s longest running data linkage agency, over more than 20 years, the Western Australian Data Linkage Branch (DLB) has developed a diverse range of sophisticated, high quality products and services based on its specialist knowledge. This paper describes DLB’s current profile. The Western Australian Department of Health (which we will refer to as the Department of Health) is in the midst of a broad functional restructure, including reforms to DLB’s data management and customer service model, geared toward further enhancing DLB’s capabilities. Those eventual changes may be discussed in a future paper.

To avoid ambiguity, at least for the purposes of this paper, these general definitions are used:

- Demographic data – the subset of administrative data used to perform the data linkage process. This typically includes identifying information such as name and address.
- Service data – the subset of administrative data that describes the underlying context of a record such as diagnosis and procedures. May also include some broad demographic information necessary for analysis (e.g. age and sex).
- Data linkage – The technique for connecting data that are thought to relate to the same person, family, place or event [1], noting this paper primarily uses the term to describe the former. This process is performed using only the demographic data.
- Linkage key – A unique group identifier assigned records that have been linked on the basis of persons, places, families or events.
- Linked data – Used both generally to describe the concept, and more specifically to refer to a collection of service data from one or more sources with linkage keys, provided for an approved request.
- Data Steward – The person legally accountable for the use and disclosure of a data collection.
- Data Custodian – The person responsible for day-to-day decisions concerning the use and disclosure of a data collection, consistent with the directions of the data steward.

## Population setting

Western Australia (WA) covers approximately a third (2.5M square kilometres) of Australia’s total landmass and 10% (2.6M people) of its population [2]. WA’s health system comprises both public and private health services, including public-private partnerships. The Department of Health is responsible for the overall management, performance, and strategic direction of the WA public health system to ensure the delivery of high-quality, safe, timely and accessible health services [3].

## DLB functions and structure

DLB is part of the Department of Health and is best known for building and operating the WA Data Linkage System (WADLS), which provides a secure and enduring data linkage infrastructure. Prior to the establishment of the WADLS, health and other administrative datasets were stored in disparate systems with little to no interconnectedness. Joined up analysis of this data was highly impractical or impossible to perform, therefore limiting the potential value of the data. The WADLS met this challenge and enables the creation of integrated datasets spanning multiple sources, which are used for approved activities such as policy development, service planning and evaluation, quality improvement and research. From its modest beginnings in 1995, the WADLS now ranks among the most comprehensive, high quality and enduring linkage systems worldwide. The WADLS spans approximately 60 data collections, representing over 115 million linked records, some dating back to as early as 1945. Health-related linkages form over half of the WADLS. DLB continues to incorporate new data collections into the WADLS, the growth of which is a focal point of the new reforms.

DLB also manages a range of services to facilitate access to linked and value-added data. This includes a refined Application for Data process and the development of innovative data delivery mechanisms (described later in this paper).

DLB comprises 19 staff across five teams aligned with the core functions described in Table 1.

In addition to its core functions, DLB has a strong track record for innovation, setting a number of successful precedents, and pioneering important pieces of work in collaboration with government, industry and academia (See Table 2). 

## Operating Model

DLB pioneered the application of the separation principle [4], which requires the separation of a person’s demographic data from their service data. It requires that those who have access to an individual’s identity do not have access to their service data and vica versa. It also helps ensure the minimum amount of information is used for the purposes of linkage and creation of linked data. This is a vital consideration, since most population health research facilitated by DLB occurs under a waiver of consent, consistent with the NHMRC National Statement [5].

The need for separation also extends to the applicant, especially in cases where they have access to identifiers. Over time, there has also been a need to extend and modify the separation principle to allow for unavoidable practical situations, for example:

- Applicants are treating physicians and therefore can intuitively recognise the service information of their patients (even where the data has had identifiers removed);
- The project depends on identifiable information to collect additional service data that cannot be sourced any other way (e.g. a review of medical notes for details that are not contained in the administrative data sourced from the hospital discharge summary); and/or
- The project team is particularly small, or where certain personnel (e.g. a PhD student) must perform a certain proportion of the work.

Situations like these may be solved using temporal separation, which permits an authorised individual access to both identifying information and service data, but prohibits simultaneous access.

### Consent model

Linkage activities in WA are not currently governed by specific state privacy law and frequently occur without informed consent. This gives rise to complex governance structures that seek to balance personal privacy with potential public benefit through secondary data use. The Government of Western Australia is developing privacy and responsible data sharing legislation and has recently published a discussion paper for public consultation [6], to which the Department of Health will contribute feedback regarding legislative enablers for data linkage and access to linked data.

Of the applications for linked data received by DLB in 2018, approximately 20% were fully consented, 75% required a waiver of consent for all data, and the remaining 5% had a combination of expressed consent and waiver of consent or the consent issues are awaiting clarification.

Decisions about waiver of consent are made by a fully constituted Human Research Ethics Committee (HREC) that is registered with the National Health and Medical Research Council's (NHMRC’s) Australian Health Ethics Committee (AHEC). DLB uses this information to inform options for release of data, and data custodians use it to inform approval for data release. A fully consented study may allow a less stringent process of separation to be approved, or ease data custodians’ concerns about the release of potentially or fully re-identifiable data.

### Governance, legislation and management

At present, there is no unifying legislation in WA to underpin the sharing of personal data, including for linkage purposes. The data linked by DLB is sourced within the WA Health System as well as via a range of other government and non-government agencies, and is governed by associated legislative and policy frameworks. The Department of Health effectively navigates a network of rules, relationships and decision-making protocols (see Table 3) to benefit all stakeholders while protecting confidentiality.

DLB’s “infrastructure linkages” (i.e. those undertaken irrespective of any project requirements, to ensure links are available upon request) are underpinned by data sharing agreements and a single, overarching HREC approval. This allows a streamlined approach to governance of the WADLS infrastructure, decoupled from the governance and time constraints of linked data applications.

## Privacy by design

DLB employs a suite of measures, in addition to those detailed above, to safeguard the confidentiality of personal data. These are documented elsewhere [7] and include physical access restrictions, criminal record checks, policy education, technological protections, data screening, encryption, secure transfer protocols and pre-publication review.

## Funding 

A linkage system the size and complexity of the WADLS comes with significant capital and operational costs, including workforce, equipment, software, and secure facilities. Additionally, the natural fluctuations in the demand for linked data make resourcing requirements difficult to predict ahead of time. The Department of Health funds DLB’s core operations. From time to time increased demand for linkage services are required, however the time required to convert additional funding into linkage capacity can create a tension between supply and demand. Expansion or changes in the WADLS infrastructure (e.g. addition of datasets) can raise similar tensions.

DLB operates a cost recovery model for non-Department of Health data requests, as well as new linkages of non-Health datasets into the WADLS infrastructure. Charging formulas are routinely reviewed to ensure sustainability and fairness, and these will be revisited once the restructure is fully implemented, to reflect DLB’s new financial position.

## Architecture and information technology

DLB maintains its own hardware, separated from the Department of Health’s network and IT support by an independent firewall. This arrangement recognises the value and sensitivity of the WADLS and permits DLB to maintain its technical architecture, software products and security systems with maximum flexibility.

The original environment was SPARC (Scalable Processor Architecture, Reduced instruction set Computing; an architecture originally developed by Sun Microsystems and Fujitsu) running Solaris and Oracle database, and while some of this style of hardware and the Solaris operating system (OS) are still in use, DLB has migrated to PostgreSQL and is moving toward Intel architecture. DLB’s strategic direction is to remove vendor dependencies and improve value for money by shifting toward generic hardware, Linux OS, and PostgreSQL database.

Much of DLB’s software is developed in-house to meet specific functional requirements. This includes linkage applications, the family connections system, data extraction scripts and a variety of other tailored data handling tools. Legacy geocoding software was developed in-house, but replaced in 2016 with an off-the-shelf product. DLB’s linkage software, including its history, drivers for change, and a description of its benefits and limitations can be found in a recently published article [9].

## Data linkage

DLB uses an enduring probabilistic linkage model. Each received dataset adds incrementally to the WADLS. This allows continuous linkage improvements with relative stability of links over time (i.e. they are not periodically re-created from scratch). Wherever practicable, data is processed, cleaned, and stored in its original structure to minimise anomalies introduced by modifying that structure [10].

The third generation of DLB’s linkage software “DLS3” recently entered production [9]. In contrast with DLB’s previous sequential/iterative approach, DLS3 is designed to incorporate new datasets into a dynamic infrastructure and allows multiple matching strategies to run concurrently. The results are combined and de-duplicated to both minimise the number of clerical reviews to be undertaken, and to also present a more holistic picture of how a record meshes with the WADLS.

DLB has a number of automated and manual processes designed to maintain and improve linkage quality [11]. DLB links a number of datasets almost exclusively for their positive contribution to linkage quality, two examples being marriage registrations (which capture many changes in surname) and drivers’ licences (which carry a history of residential addresses).

Holistic WADLS statistics and Dataset Quality Statements have been routinely produced for some time [11], and more recently work has commenced on producing standardised linkage reports at the completion of each linkage in DLS3. These will provide valuable detail to applicants regarding outcomes for project-related linkages. Reports for routine linkages will allow a trend analysis of linkage quality over time to inform continuous improvement.

## Data sources

DLB undertakes most infrastructure linkages on a consistent schedule (monthly, quarterly or annually). Some infrastructure linkages are more sporadic, depending on resourcing and data availability.

As shown in Table 4, DLB’s current linkage infrastructure comprises 44 recently or routinely linked data collections. Excluded from this table are a number of historical infrastructure linkages for which the existing links are available but the data has not been recently updated. DLB also completed around 500 project-specific linkages since its inception, 55 of which were performed during 2017 to 2019. Some of these are deemed infrastructure equivalent due to their scale, complexity or maintenance requirements; the latter have been included in the table. The new Mental Health Information Data Collection (MIND) and the Non-admitted Patient Activity and Wait List (NAPAAWL) data collections are two recent and valuable additions to DLB’s linkage infrastructure.

The addition of new datasets to the WADLS is typically negotiated based on their known or anticipated level of demand. Data custodians typically perceive the greatest risk lies in the disclosure of personal identifiers, usually a necessity for linkage to occur. In a reasonably mature linkage system such as the WADLS, it can be assumed that the vast majority of these identifiers already exist within the system. The risk factor is more often in the additional information added to the linkage system, such as identifying a person has a particularly sensitive characteristic (e.g. someone with an infectious disease).

The time and resourcing requirements to link demographic data and make associated service data available are often underestimated. Many data collections enforce their own quality assurance and review processes that can extend the timeframe for data to become available for linkage. Beyond this, problems with the linkage readiness [12] of demographic data often need to be addressed. These include issues of completeness and quality, consistency between updates, and the stability of unique record identifiers. Alternatively, data providers may be able to provide data for linkage, but then experience lengthy delays delivering the service data extracts (e.g., due to resourcing constraints). Many of these problems can put data linkage on hold, delay requests, or invalidate work already completed. DLB finds these issues are best mitigated when data providers treat linkage as a mutually beneficial ongoing partnership that requires regular communication and troubleshooting. Clear process documentation for on-boarding new and updated datasets and thorough acceptance testing are highly beneficial in ensuring that problems are captured early. Routine review and reconciliation processes can also assist in identifying data discrepancies that have “slipped through the net.”

## Linkage keys

The term ‘linkage key’ is used varyingly by different linkage agencies. In some contexts, it has been used to describe the set of identifiers used to match with other data [13]. In DLB’s case, the term refers to a unique group identifier.

Demographic data is stored within DLS3 as a collection of *“demogs”*. A *demog* consists of one or more unit-level data records. A *record identifier* (DLB’s clients may know this as the LPNO) is assigned to each *demog*. These identifiers are unique across data collections, but stable within them [14].

DLS3 compares *demogs* to determine the potential for a match. However, it does not store any resulting linkage as a relationship between two *demogs*. Instead, it combines the potential match with all the potential matches for other *demogs* already associated together, and then adds them to the list considered to belong to the same person. This list is called a *chain*. Currently, there are >4.4 million *chains* in the WADLS (excluding chains of only one record). The median chain length is 27, the longest exceeds 10,000 records.

*Chains* are stored in an independent ‘Links’ table, comprising *chain* identifiers and pointers to their associated demographic data. This is DLB’s equivalent of what is sometimes referred to as a ‘Master Linkage Key’. Adding a record to a *chain* triggers the automatic validation process within DLS3 that reviews the *chain* for unlikely or impossible record combinations [9, 15^15^].

A *chain* can be identified by any *record identifier* within the *chain*. However, one is chosen by a heuristic that prioritises those from sources that have historically remained most stable in *chains*. These are generally from events at the very beginning of a person's life (e.g. a birth record). This *record identifier* is called the *root*, and for most user-visible purposes it is the chain identifier.

The *root* is the external presentation of a linkage key. This structure is a legacy from the previous linkage system and has been maintained for its familiarity to pre-existing applicants and to smooth transition when data updates are requested. While DLS3 supports this structure, it is not constrained by it. Any record, sub-type and/or linked group can be identified using native DLS3 fields alone.

Project specific linkages that are not deemed infrastructure-equivalent are performed without traditional *record identifiers* being assigned. DLS3 performs all the same cleaning and linkage functions, and identifies the *chain* to which the record would be added, but does not add the record to the *chain*. This allows the full benefits of DLS3 to be realised and ensures the linkage can be accessed again if required, but avoids the unnecessary overhead of ongoing maintenance.

### Service data extraction

Most applications for data require a tailored extraction that meets specified parameters, including cohort selection criteria (e.g. based on diagnoses), date bounds and a defined list of variables for each dataset being requested.

The standard extraction process [16] in DLB has five steps:

1. Identify the study population(s),
2. Extract the associated linkage keys,
3. Attach the approved service data,
4. Perform standardisation and quality checking, and
5. Securely release the data to the approved recipient.

DLB developed the Custodian Administered Research Extract Server (CARES) [17], a centralised repository of service data from participating data collections and a suite of associated extraction tools. CARES was developed to overcome inefficiencies with the traditional linked data extraction process, where the task of extracting service data was distributed among every one of the participating data collections (hospital, emergency, cancer, death, etc.), each with their own data stores, tools, output formats and competing priorities for resourcing. Data custodians using the CARES system are required to provide routine updates of their service data for CARES. CARES improves the efficiency of the extraction process by allowing the previously disparate tasks to be performed by one person in a standardised environment. CARES has been embraced by a number of data custodians for the reduction in workload, although subsequent cost shifting to DLB has been a consequence. DLB are considering ways to further improve the CARES model by reducing or eliminating some of the interdependencies between CARES, WADLS and the source Data Collections.

### Access to data

Traditionally DLB delivers tailored extracts to applicants who have met the required data, ethical and research governance requirements. These extracts contain the minimum data necessary to meet the analytical requirements of the project. Historically, this approach has suited most applications for data received by DLB. However, as the breadth and complexity of linked data requests increase, DLB is considering alternatives to this traditional approach.

Data custodians have an unparalleled knowledge of the data collections they manage that adds value when reviewing requests for linked data. This expertise is built through their day-to-day experience as area managers who handle a range of responsibilities related to their data collection. They provide valuable services associated with linked data requests, including application review, data extractions and pre-publication review. As linked data requests have increased in number and complexity, the duties of data custodians have become more demanding. The Department of Health reforms will target improvements to the effective support for this critical function.

Requests for population-wide datasets to be used by multiple personnel for a variety of projects (sometimes unspecified at the time of application) are increasingly common. The current policy and legislative framework does not always provide a clear path to facilitating these requests. Navigating them case-by-case is onerous and can impact feasibility, cost and timeframes. Novel approaches to data governance and technical innovations (such as secure access laboratories) have delivered some progress in this space. DLB are investigating new ways to achieve practicable and cost-effective solutions that mitigate risks while encouraging the fullest use of administrative data for the public good. This includes review of governance models for linked data repositories, development of a Department of Health linked data warehouse and associated secure access environments, and revisions to the Application for Data process to better accommodate the full spectrum of requests. Additionally, the Department of Health is contributing to a range of state and national conversations on data sharing, privacy and associated policies and legislation. Developments in this space will have considerable impact on data linkage capability in Western Australia and nationally.

### Noteworthy outputs

DLB has conducted two reviews of linked data projects and their outputs that collectively cover the period 1995 to 2014. “Outputs” were defined as “a publication produced by the researchers using linked data provided by the DLB”. In addition to outputs self-reported by the researchers, DLB also searched for outputs containing acknowledgement of DLB as a source of linked data. Together these scans revealed a total of more than 2350 publications, spanning over 800 projects.

The results of linked data projects have been used in a variety of ways, including:

- The planning and implementation of health services (dialysis, palliative care, dental, aboriginal health, vaccinations, chronic disease, etc.)
- Policy development (breast cancer, vaccinations, vehicle safety, aged care, occupational safety, etc.)
- The establishment of new data collections (developmental anomalies, intellectual disability)
- The development and refinement of new data linkage processes (cross-jurisdictional linkage, privacy preserving record linkage)
- Evidence-based clinical evaluation (Hepatitis C testing, opiate prescriptions, etc.)

An independent national review carried out in 2015 [18] found that 51% of 629 papers identified to have used Australian linked hospital data had received data from WA, contrasting with WA’s 10% share of the national population. Fewer than ten of the identified papers were published prior to DLB’s establishment in 1995.

## Discussion

Data linkage is a complex, multi-stakeholder process, challenging to understand and even more so to perform. DLB’s operations are perceived by some as a black box, leading to misperceptions of the factors affecting (1) the linkage of data, (2) access to linked data; and (3) the analysis of linked data. Expertise in one of these areas does not necessarily translate to the others. Challenges may relate to funding, governance, oversight, protocol or technology. Some of these challenges are internal to DLB and others fall in the remit of other stakeholders to fix, or a combination of the two. Siloed thinking risks misunderstanding the root cause of challenges faced by other parties. DLB recognises that different parties provide valuable and complementary perspectives and welcomes opportunities to collaborate. To facilitate these discussions, the Department of Health is seeking to establish a Data Linkage Stakeholder Advisory Committee, drawing input from a range of government and non-government participants.

The lack of cohesive WA legislation that explicitly and unambiguously enables data sharing, data linkage and associated data release has driven a proliferation of governance practices (sometimes overlapping) and a lack of clarity about data sovereignty. Delayed or ad-hoc decision-making complicates and further obstructs data sharing for linkage and release of data to applicants. Having common privacy, data classification, risk assessment and data sharing models, alongside the provision of associated technical solutions, will address some challenges facing linkage agencies and applicants alike.

Rational consideration of the sensitivity of data is central to the continued progress of data linkage at all levels. The Australian Government is currently consulting with a wide range of agencies regarding its new data sharing legislation. DLB is hopeful that inclusion of data linkage agencies in this consultation will ensure a result that enables rather than constrains their activities, including in cross-jurisdictional environments.

To ensure a successful outcome, any smoothing of data sharing pathways must be coupled with strong and enduring partnerships between data providers and linkage agencies. High quality linkages, consistent update schedules and timely access to data are more likely when all stakeholders view linkage and the associated technical and governance considerations as a collaborative effort. Further value can be realised when data providers routinely use linkage to improve the quality and usability of their own data stores.

Success breeds growth in demand and challenges may arise when the requirements for commensurate growth in data stores, processing power, operational throughput and personnel are limited by funding. For example, linked outpatient data has long been sought after, but with a volume considerably greater than that of DLB’s next largest data collection, the Non-admitted Patient Activity and Wait List (NAPAAWL) linkage has only recently become viable thanks to the rollout of DLS3. Even so, DLB must still grapple with server resourcing considerations to handle the collection’s sheer volume. DLB is watching emerging technology with keen interest, for example, the possibility of leveraging secure cloud services to improve processing power and efficiency.

Under the Department of Health’s restructure, important steps are being taken to remedy DLB’s historical challenges with respect to balancing linkage supply and demand. DLB’s experience and lessons learned have shown that funding models for linkage agencies should be approached with considerable foresight. Acceptable funding during the start-up phase may not translate well to longer term funding needs. A reliance on grants, charitable funding, and cost-recovery may hinder the progress toward strategic goals in favour of short-term outcomes. A proliferation of temporary contracts allows teams to be restructured to best meet varying demand; however this comes at the expense of job security and may result in the loss of skilled staff.

Mature linkage agencies face the challenge of balancing stability with adaptability in an environment of variable but increasing demand. Agencies employing a fixed-funding model may experience limited ability to adapt, potentially limiting their opportunities to grow. However, agencies operating under cost recovery scenarios may also face problems, since allocation of resources is harder to plan ahead of time and any increases in funding run the risk of being mistimed with operational needs or quickly subsumed by demand. 

## Conclusion

DLB is proud of its achievements, and excited to be a part of the rapidly maturing and vibrant Australian data linkage community. DLB’s culture of continuous improvement has led many of its practices, service models and innovations to be adopted elsewhere. However, such self-driven progress must be balanced with well-meaning external recommendations and the constant demand to deliver data for applicants as quickly as possible, an experience almost certainly not unique to DLB. The Department of Health is committed to building on DLB’s historical achievements and to continue its pre-eminent standing in the global linkage community. With this, alongside effective collaborations, technological advances and developments in enabling legislation, DLB aspires to a future linkage landscape that is sustainable, high quality, efficient and safe.

## Contributions

SH drafted the original manuscript with input and critical feedback provided by TE, AM and JA.

## Ethics statement

The data custodian of the WA Data Linkage System determined that no Ethics approval was required for this publication.

## Tables

**Table 1: Data Linkage Branch teams:** 

Team	Function
Linkage Team	The DLB Linkage Team is responsible for the day-to-day operation of the WADLS including the receipt, processing, linkage, and extraction of health-related and other datasets, as well as value-adding processes such as those related to geocoding and family connections. Overseen by the Team Leader, Linkage and Systems.
Client Services Team	The Client Services team assists applicants for linked data through the application and approval process that involves a variety of legal, ethical and governance arrangements. This process includes interactions with applicants to understand their linked data needs, facilitating feasibility assessments and approvals, managing data requests, preparation and delivery. This involves considerable negotiation with applicants, data custodians and staff undertaking data extraction to ensure best practice protocols are followed and information is delivered in an agreed and timely manner. Overseen by the Team Leader, Client Services and Data Delivery.
Data Delivery Team	This DLB team is responsible for the maintenance and regular loading of supplied datasets into the Custodian Administered Research Extract Server (CARES). CARES is a centralised repository of service data from a number of participating collections and is described in detail elsewhere [4]. This team is also responsible for preparation of approved bespoke linked data extracts using CARES, integration of other datasets and performing quality assurance on linked data prior to release to applicants. Overseen by the Team Leader, Client Services and Data Delivery.
Systems Team	This DLB team is responsible for the development of DLB’s bespoke linkage software (called DLS3) [9], maintenance of DLB’s servers, and the support of DLB-specific IT infrastructure and its users. Overseen by the Team Leader, Linkage and Systems.
Strategy and Leadership Team	In addition to supporting the teams above, this team undertakes strategic planning, maintains expert awareness of linked data methods and protocols, collaborates with other stakeholders, performs delegated data custodian duties, and represents the Department of Health in relevant state and national forums

**Table 2: DLB Innovations, Precedents, and Pioneering collaborations:** 

Project/Initiative	Description
Researcher training courses	Conducted three to four times annually to prepare applicants to navigate the linked data application and approvals processes.
Project complexity	Development of an algorithm to measure the complexity of linked data projects [19].
Application process	A standardised and modularised data application process [20].
Family Connections System	Creating genealogical linkages using data from the Registry of Births, Deaths and Marriages, and the Midwives Notifications System [21].
Aboriginal and Torres Strait Status Flag	Creating and validating an algorithm to derive Aboriginal and Torres Strait Islander status using information from multiple data collections [22].
Dataset Quality Statements	The quality and completeness of the records that are provided to DLB influences the quality of the links that can be made between them. Data Quality Statements provide insight into the characteristics of the many datasets DLB holds, to help users of linked data understand some of the challenges faced in linking them together [15].
Routine geocoding	Matching address information from linked administrative data collections to reference data, to enable its use for spatial mapping and other applications (e.g., enhancements with national measures of social and locational disadvantage).
CARES	Creating the CARES [17] infrastructure to improve timeframes by streamlining the data delivery process and reducing the burden on data custodians. It is maintained and operated by the Data Delivery Team.
DLS3	Designing in-house data linkage software (DLS3) [9] that achieves an efficient, adaptable and scalable solution to DLB’s evolving linkage needs. It is developed and maintained by the System’s Team.
Data Custodians and Stewards meetings	Regular Data Custodian and Steward meetings that seek practical solutions to various hot issues that impact the availability and timeliness of linked data, mostly related to policy, legislation, other regulatory developments and resourcing.
Road Safety Data Linkage Infrastructure Project	A partnership with the WA Road Safety Commission to link a number of relevant data sets to enable enhanced road safety research [23].
Target 120 Project	A State Government election commitment to develop linked data infrastructure that improves the understanding and outcomes for young offenders and their families [24]. DLB oversees the health-related component of a state-level distributed linkage model, and contributes linked health data and family connections to the resulting service database, the Social Investment Data Resource (SIDR).
Renal Demand Modelling Project	An ongoing Department of Health service planning initiative to achieve a more accurate, valid and reliable data set for renal service planning across WA.
Developmental Pathways Project	Inaugural linkages of a number of social services data collections, enabling linked data analysis to “investigate risk and protective factors that lead to differences in developmental outcomes for children and youth” [25].
Department of Health core business	The use of linkage keys for non-research core Department of Health business, including public health monitoring, service evaluations, modelling, waitlist management and development of key performance indicators.

**Table 3: Governance of the use of Linked Data:** 

Governance of the use of Linked Data

The Department of Health’s governance of data, including linkage keys, is consistent with the collection, access and disclosure requirements embodied in a variety of legislation, policy frameworks and regulations.
The Director General of the Department of Health delegates these data responsibilities to data stewards, who are assisted by data custodians, noting that the latter are unparalleled subject-matter experts in the collection, management and interpretation of health data.
All data release must be authorised in line with the Director General approved policies.
Where personal health data is used for research, it requires ethical approval from the Department of Health’s NHMRC accredited HREC and research governance approval via the Research Development Unit.
Use of DLB products and services, including linkage keys, requires authorisation. Data custodians and DLB are jointly responsible for ensuring linked data requests are technically and logically feasible, which at times includes escalation to the relevant data steward(s).
DLB coordinates this activity using a streamlined application review process [20] that includes a preliminary assessment of an application’s feasibility and the provision of expert advice. DLB will typically also coordinate the draft application review process on behalf of non-Department of Health data custodians.
DLB’s application process is independent from the HREC, but assists applicants with their preparation to submit their ethics application. Feedback from the HREC indicates that requests for linked data are better prepared coming via DLB’s application process than other avenues.
Importantly, the Department of Health respects that non-Department of Health data custodians have their own independent processes that the Department of Health has no authority to change, prescribe, override, or hasten.
Differences in policies and legislation between jurisdictions must be considered when national or cross-jurisdictional linkages are featured.

**Table 4: Summary of Data Linkage Branch linkage infrastructure 2017 to 2019:** 

Data Collection		Frequency
1.	Emergency Department Data Collection	Weekly and Monthly
2.	Hospital Morbidity Data Collection	Monthly
3.	Midwives Notifications System	Monthly
4.	WA Cancer Registry	Monthly
5.	Mental Health Information System	Monthly; concluded
6.	MIND Mental Health	Monthly
7.	Registrar General - Birth Registrations	Monthly
8.	Registrar General - Death Registrations	Monthly
9.	Registrar General - Marriage Registrations	Monthly
10.	Elective Surgery Waitlist	Monthly
11.	Non-Admitted Activity & Wait List	Monthly
12.	WA Electoral Roll	Quarterly
13.	Cause of Death Unit Record File	Annual
14.	Department of Transport Drivers’ Licences	Annual
15.	Main Roads WA Crashes	Annual
16.	Insurance Commission WA	Annual
17.	Trauma Registry	Annual
18.	Breastscreen WA	Annual
19.	Monitoring of Drugs of Dependence System	Annual
20.	WA Register of Developmental Anomalies - Birth Defects	Annual
21.	WA Register of Developmental Anomalies - Cerebral Palsy	Annual
22.	WA Notifiable Infectious Diseases Database	Annual
23.	Health and Wellbeing Surveillance System	Annual
24.	iPharmacy	Annual
25.	WA Cervical Cancer Prevention Program	Annual
26.	Australian Early Development Census	3-yearly
27.	Australia and New Zealand Dialysis and Transplant Registry	Project-specific; annual
28.	Pathwest	Project-specific; annual
29.	Clinipath	Project-specific; annual
30.	Clinical Labs WA	Project-specific; annual
31.	St. John of God Pathology	Project-specific; once-off
32.	Perth Pathology	Project-specific; once-off
33.	Dental Health Services	Project-specific; once-off
34.	Oral Health Care WA	Project-specific; once-off
35.	iPharmacy (Sir Charles Gairdner Hospital)	Once-off
36.	ASCribe	Once-off
37.	Home & Community Care	Once-off
38.	Aged Care Assessment Program	Once-off
39.	Intellectual Disability Exploring Answers Database	Upon request
40.	St John Ambulance	Upon request
41.	Silver Chain WA	Upon request
42.	Royal Flying Doctors Service	Inaugural linkage only; ongoing status TBD
43.	Child Development Information System	Inaugural linkage only; ongoing status TBD
44.	Brightwater Care Group	Inaugural linkage only; ongoing status TBD
